# Prospective comparison of indwelling cannulas drain and needle aspiration for symptomatic seroma after mastectomy in breast cancer patients

**DOI:** 10.1007/s00404-019-05396-2

**Published:** 2019-11-28

**Authors:** Xiufeng Wu, Yiping Luo, Yi Zeng, Wei Peng, Zhaoming Zhong

**Affiliations:** 1 Department of Breast Surgical Oncology, Fujian Medical University Cancer Hospital & Fujian Cancer Hospital, No. 420 Fu Ma Road, Fuzhou, 350014 Fujian People’s Republic of China; 2 Department of Anesthesiology, Fujian Medical University Cancer Hospital & Fujian Cancer Hospital, Fuzhou, 350014 Fujian People’s Republic of China; 3 Department of Clinical Laboratory, Fujian Medical University Cancer Hospital & Fujian Cancer Hospital, Fuzhou, 350014 Fujian People’s Republic of China; 4 Department of Ultrasound, Fujian Medical University Cancer Hospital & Fujian Cancer Hospital, Fuzhou, 350014 Fujian People’s Republic of China

**Keywords:** Breast cancer, Seroma, Aspiration, Indwelling cannulas

## Abstract

**Aims:**

Postoperative seroma is the most frequent sequelae after mastectomy and axillary surgery with no optimal regimens for seroma resolution recommended in routine clinical. Indwelling cannulas with needle and catheter have been widely used in long-term medication therapies, but evidence of indwelling cannulas in seroma management after mastectomy is lacking. The purpose of this study is to evaluate the feasibility of indwelling cannulas in seroma management after mastectomy.

**Methods:**

Patients who underwent modified radical mastectomy (MRM) and developed symptomatic seroma after removal of the drains between August 2017 and December 2018, were randomized into two groups either indwelling cannulas drain of seroma (Group A) or needle aspiration of seroma (Group B). We prospectively compared the number of visits for seroma, the time from removal of the drain to the final seroma resolution and the cost between the methods.

**Results:**

A total of 860 patients underwent MRM between August 2017 and December 2018, among which 86 patients who developed symptomatic seroma after removal of the drains, were randomized into two groups either Group A or Group B. The number of visits for seroma in Group A was 2.35 ± 0.69 times, which was less than those in Group B (4.86 ± 1.06 times). Similarly, the time of drain removal to final seroma resolution in Group A was 4.65 ± 0.78 days, which was shorter than 7.09 ± 1.54 in Group B. In Group A, the total mean cost per patient (25.81 ± 7.71 RMB) was less than the total mean cost per patient (49.30 ± 9.85 RMB) in Group B. Cost savings were noted with using indwelling cannulas in seroma management.

**Conclusion:**

It is feasible to drain indwelling cannulas drain for postmastectomy seroma, with less visits for patients, rapid seroma resolution and less cost. Indwelling cannulas can be an efficient, cost effective solution to treat symptomatic seroma after breast surgery.

## Introduction

Seroma is the most frequent sequelae occurring after mastectomy with an incidence ranging from 3 to 85% [1–3]. Seroma may increase the risk for infection leading to surgical wound complications and may also significantly affect treatment by delaying adjuvant therapy, resulting in less effective breast cancer treatment [4, 5].

The most frequently used techniques for seroma treatment include drain replacement [6] and multiple needle aspiration depending on seroma size, symptoms, and patient preference. However, both methods have well-known limitations and risks. Drain replacement is typically used when the seroma has lasted long after multiple aspiration attempts [7]. It can be uncomfortable for patients and associated with an increased risk of infection. Similarly, needle aspiration may increase the risk of infection in addition to multiple visits resulting in waste of the hospital resources. Furthermore, needle aspiration is less aseptic and time consuming especially in a busy outpatient setting. In addition, needle aspiration does not allow complete drainage of seroma. Several groups have reported so-called seromadesis in which agents are introduced into the seroma cavity to promote adhesion of flaps to the chest wall [8–11]. However, this method can cause discomfort of patients and does not allow complete drainage of seroma in one time. Recently, Khalid et al. [12] stated a needle attached to a high vacuum wound drainage system allows efficient and rapid drainage of the seroma to dryness without multiple visits. However, this technique requires special equipment in outpatient room and can lead to pain and cost a lot for patients. Currently, there is no standardized procedure for treating established seromas. It would therefore be necessary to have a seroma management technique that is efficient and cost effective.

Indwelling cannulas (IC) with needle and catheter are widely used for long-term nutrition and medication therapies as they can remain intravenously for up to 29 days at a time [13]. Because IC does appear successful for long-term medication therapies, we begin to offer this device as an option for managing postmastectomy seroma.

The present prospective randomized study compares the number of visits for seroma, the time from removal of the drain to the final resolution and the cost between IC and needle aspiration in patients with postmastectomy seroma. The purpose of this study is to evaluate the feasibility of IC in seroma management. This is, to our best knowledge, the first demonstration of the use of IC in seroma management after breast cancer surgery.

## Methods

### Ethics statement

The study was conducted in Fujian Medical University Cancer Hospital & Fujian Cancer Hospital from August 2017 to December 2018. Patients underwent modified radical mastectomy (MRM) and developed symptomatic seroma after removal of the drains were recruited to participate in this study, which was approved by Institutional Review Board of Fujian Provincial Cancer Hospital. Written informed consent was obtained prior to study participation.

### Patients

**Fig. 1:**
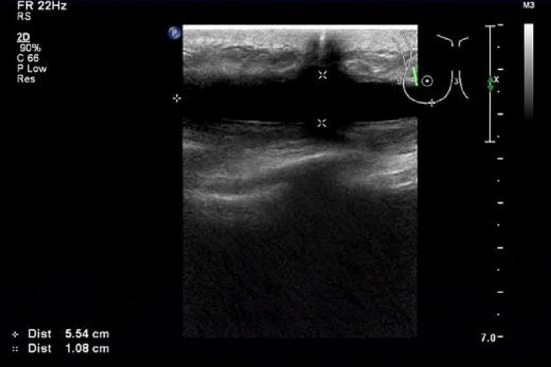
Ultrasound image of seroma after removal of the drains in a breast cancer patient who underwent MRM on the right side

The type of surgery is the most important factor for seroma production. To reduce the influence of type of operation in seroma formation, only consecutive patients underwent modified radical mastectomy (MRM) were included. Surgery was performed by the same surgical team comprising three surgeons (one senior and two resident surgeons) using a standardized technique with harmonic scalpel. Axillary dissection was done up to level I–III in all the cases. Two drains were placed in the cavities to ensure complete drainage. Drains were removed if the collected volume in 24 h was less than 50 ml. Seroma in this study was defined as a symptomatic fluctuant collection in the wound site then verified by ultrasound (Fig. 1). Patients who suffered symptomatic seroma complained distending pain in the wound site. Since seroma may increase the risk for infection leading to surgical wound complications and may also significantly affect treatment by delaying adjuvant therapy, resulting in less effective breast cancer treatment, so it is important to empty the seromas. Patients who developed symptomatic seroma were then randomised to undergo either indwelling cannulas (B. Braun Melsungen, German) drain (ICG) (Group A) or needle aspiration (NSG) (Group B) of the seroma until resolution.

### Procedures

**Fig. 2:**
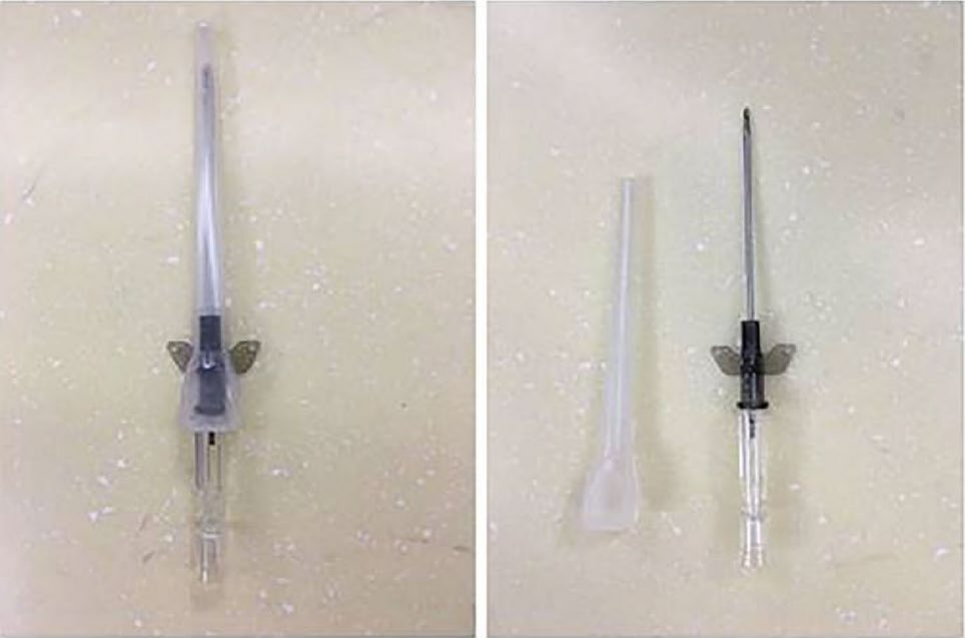
Indwelling cannulas (16-gauge)

**Fig. 3:**
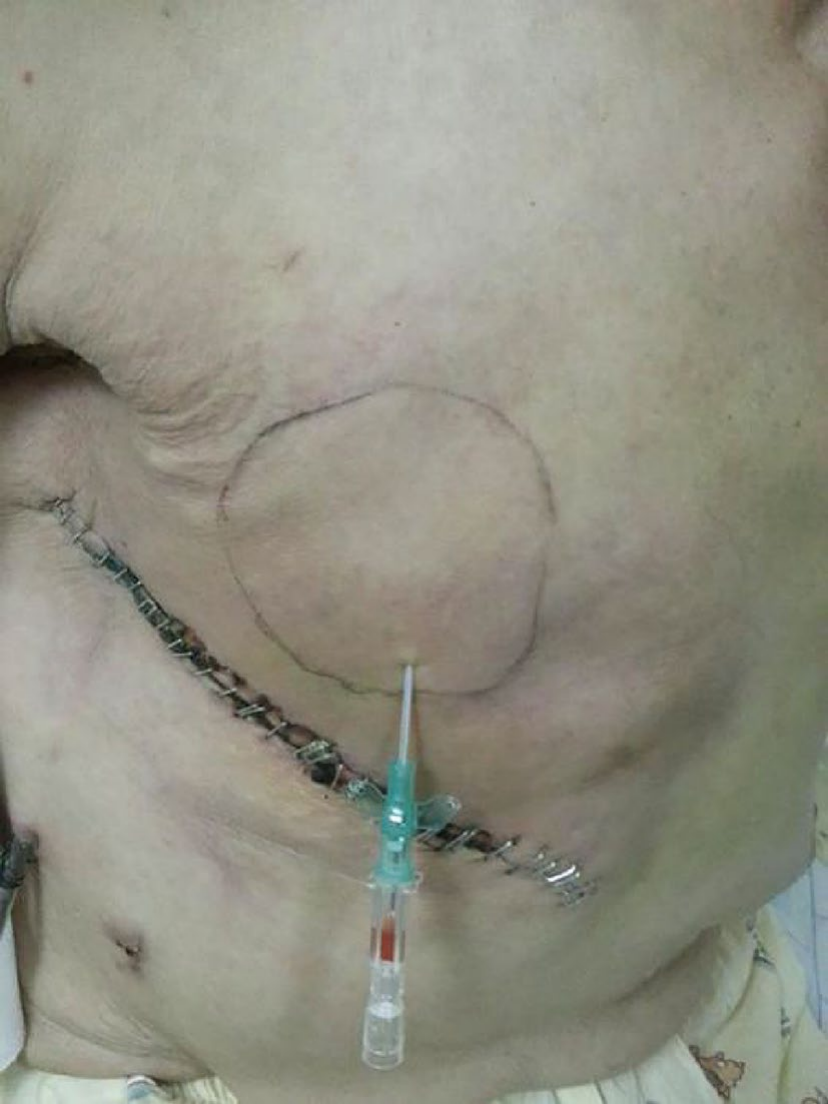
Drainage of seroma using indwelling cannulas in a breast cancer patient who suffered postmastectomy seroma after drainage removal

In ICG group, the patient was positioned by sitting on the chair, so that the seroma collection was localized by the effects of gravity. An indwelling cannula (16-gauge) (Fig. 2) needle was passed through the previous surgical scar without local anesthetic using an aseptic technique (Fig. 3). Keep indwelling cannulas in position until seroma has resolved. To eliminate dead space, external compression dressing was provided with an external compression dressing. In NSG group, patients visited doctor for hypodermic syringe needle aspiration until the amount of fluid in the last 24 h reached below 5 ml.

All procedures were undertaken on the surgical ward using an aseptic technique. The two groups were comparable in respect of age, body mass index (BMI), tumor size, number of lymph nodes removed, and number involved. The number of visits for seroma, the time from removal of the drain to the final seroma resolution and the cost were compared between two groups.

### Statistical methods used

Descriptive studies were performed with SPSS version 10 and group characteristics were compared using Student’s *t*-test.

### Results

A total of 860 patients underwent MRM between August 2017 and December 2018, among which 86 patients suffered symptomatic seroma after drainage removal. The mean volume across all patients in this series was 50 ml (20–110 ml) according to ultrasound. The 86 patients who developed a symptomatic seroma were randomized to either ICG (Group A) or NSG (Group B). Patient data are shown in Table 1. No patients developed a wound infection during treatment between two methods. No seromas recurred, either. Table 2 shows the comparative data for the two treatment groups. All seromas had resolved at the 2 week outpatient review. The number of visits was 2.35 ± 0.69 times in group A and the number of visits in group B was 4.86 ± 1.06 times. A significantly lower number of visits were observed in group A than in group B (P = 0 0.001). The time of drain removal to final seroma resolution in group A was 4.65 ± 0.78 days, which was significantly shorter than that in group B (7.09 ± 1.54) (P = 0 0.001) (Table 2).

**Table 1:** Patients and tumor characteristics

Variable	ICG (N = 43)	NSG (N = 43)	*p*
Age (years)	48.70 ± 9.33	49.05 ± 9.16	0.862
BMI	22.37 ± 2.13	21.96 ± 1.80	0.341
Tumor size (cm)	2.93 ± 1.26	2.45 ± 1.32	0.087
No of removed nodes	21.26 ± 6.48	22.53 ± 6.93	0.379
No of nodes involved	3.14 ± 3.38	3.30 ± 2.86	0.810

**Table 2:** Comparison of the two treatment methods for seromas

Variable	ICG	NSG	*p*
Mean number of visits (times/patient)	2.35 ± 0.69	4.86 ± 1.06	0.001
Time from drain removal to final aspiration (days)	4.65 ± 0.78	7.09 ± 1.54	0.001
Cost (RMB)	25.81 ± 7.71	49.30 ± 9.85	0.001

The cost of use of indwelling cannulas and needle was estimated on the basis of the material supply charge and labor cost. The material supply charge included indwelling cannulas, needle, and other associated cost. Labor cost was calculated on the basis of average nursing wages of 6.0 RMB one time. As a result, total costs of indwelling cannulas were 25.81 ± 7.71 RMB, which was less than total costs of needle aspiration (49.30 ± 9.85 RMB).

## Discussion

Seroma is the most frequent complication following breast and axillary surgery. Although seroma does not endanger life, it may cause anxiety and discomfort for the patient, even give rise to a variety of problems such as skin flap necrosis, wound breakdown, decreased shoulder mobility, and delay of adjuvant chemotherapy and radiotherapy [1, 2]. Despite efforts to prevent seroma formation, incidence rates remain high. A variety of techniques has been employed to seromas, including multiple aspiration, drain replacement, or sclerotherapy with agents [14]. These measures may be helpful in seroma resolution. However, no optimal regimens for seroma management are recommended in routine clinical.

The present prospective study has confirmed that indwelling cannulas can be used successfully for seroma management, which is efficient and cost effective. Indwelling cannulas is flexible, which is safe to be inserted into a cavity for draining off fluid without any discomfort and it can remain in place for long at a time. In addition, it is easier to place than a drain and it does not last as long and can often take multiple attempts to be successfully. No seromas have recurred using indwelling cannulas in this study. The total amount of visits for seroma in ICG group is significantly less than that in NSG in our study, which reduces patient discomfort and the workload of medical staff. In China, especially in rural areas, patients tend to take 1 day to visit doctor and are troubled with seroma requiring multipasses. In addition, the time of resolution of the seroma in ICG group is shorter than that in NSG group. The previous study has reported no significant difference in time to seroma resolution in a randomized controlled trial of daily aspirations versus aspiration as needed for patient symptomatology [15]. The possible reason for difference between our study and previous study is that persistent drain using IC other than daily aspirations in our study would keep the wound cavity dry and allow the wound flaps to adhere to the chest wall preventing reaccumulation of fluid, resulting in a more rapid resolution.

Without health insurance coverage in China, especially in rural areas, the cost for seroma treatment is one of the concerns for patients. In our study, total costs of indwelling cannulas are less than total costs of needle aspiration, which means patients in IC group require less use of hospital resources with lower cost compared with patients in NSG group.

In our study, the seroma incidence after modified radical mastectomy is 10%, which is comparable with the previously published study. The use of harmonic scalpel during surgery has been shown to decrease seromas formation [16]. The use of harmonic scalpel in our study is associated with relatively low incidence of seroma formation.

In summary, this prospective study has demonstrated the feasibility of using IC for seroma management following breast surgery. IC may be a safe and cost effective solution to treat symptomatic seroma after breast surgery and is particularly useful in busy outpatient setting. Further evaluation of this management strategy and patient satisfaction is required.
